# Pre-Processing Method to Improve Cross-Domain Fault Diagnosis for Bearing

**DOI:** 10.3390/s21154970

**Published:** 2021-07-21

**Authors:** Taeyun Kim, Jangbom Chai

**Affiliations:** Machine Diagnosis Laboratory, Department of Mechanical Engineering, Ajou University, Suwon 16499, Korea; lonoa91@ajou.ac.kr

**Keywords:** bearing fault diagnosis, cross-domain fault diagnosis, domain adaptation, signal processing, transfer learning

## Abstract

Models trained with one system fail to identify other systems accurately because of domain shifts. To perform domain adaptation, numerous studies have been conducted in many fields and have successfully aligned different domains into one domain. The domain shift problem is caused by the difference of distributions between two domains, which is solved by reducing this difference. Source domain data are labeled and used for training the models to extract the features while the target domain data are unlabeled or partially labeled and only used for aligning. Bearings play important roles in rotating machines, so many artificial intelligent models have been developed to diagnose bearings. Bearing diagnosis has also faced a domain shift problem due to various operating conditions such as experimental environment, number of balls, degree of defects, and rotational speed. Cross-domain fault diagnosis has been successfully performed when the systems are the same but operating conditions are different. However, the results are poor when diagnosing different bearing systems because the characteristics of the signals such as specific frequencies depend on the specifications. In this paper, the pre-processing method was used for improving the diagnosis without prior knowledge such as fault frequencies. The signals were first transformed to a common pattern space before entering the models. To develop and to validate the proposed method for different domains, vibration signals measured from two ball-bearing systems (Case Western Reserve University datasets and Paderborn University datasets) were used. One dimensional CNN models were utilized for verification of the proposed method and the results of the models using raw datasets and pre-processed datasets were compared. Even though each of the ball-bearing systems have their own specifications, using the proposed method was very helpful for domain adaptation, and cross-domain fault diagnosis was performed with high accuracy.

## 1. Introduction

Rotating machines play a very important role in manufacturing plants. Among the many parts of rotating machines, bearings have a significant impact on the operation of rotating machines. Failures of electro-mechanical drive systems and motors are caused by rolling bearings with high probability [1]. Therefore, bearing diagnosis is important in order to use rotating machines safely and studies on this has been actively conducted. There are several open datasets which are conducted in various operating conditions such as Paderborn University datasets (PU) [1] and Case Western Reserve University datasets (CWRU) [2]. Smith et al. proposed some signal processing methods that make the characteristics of faults show more clearly using CWRU datasets and interpreted the results using the fault frequencies [3]. However, as the processing speed of computers and the size of data that can be stored increase, diagnostic studies using data-driven methods have rapidly increased. Artificial intelligence algorithms for bearing diagnosis such as random forest, Bayesian network, support vector machine, neuro-fuzzy, and artificial neural network have been conducted [4]. In addition, deep learning models such as convolution neural network (CNN), stacked autoencoder, and deep belief network have also been applied to bearing diagnosis [5].

CNNs are good at extracting features, and therefore many studies on artificial intelligence (AI) models with CNNs for diagnosing bearing faults have been conducted [6,7,8,9,10,11,12,13,14,15,16,17,18,19]. Janssens et al. confirmed that the feature learning model based on convolutional neural networks for bearing fault diagnosis could outperform the classical approach which uses engineered features and a random forest classifier [6]. Peng et al. developed a deeper 1D CNN based on Resnet and enhanced the anti-noise ability by introducing a wide convolutional kernel and dropout [7]. Peng’s model showed great performance even for data with strong noise. Huang et al. proposed using filters in different lengths with a CNN so that more useful information could be extracted [8]. Wen et al. transformed the vibration data into an image and developed a new CNN model based on Lenet-5. The performance of Wen’s model outperformed other models such as support vector machines, conventional CNNs, and artificial neural networks [9]. Wang et al. also visualized the vibration signal from a bearing by symmetrized dot pattern (SDP) and developed a model based on the squeeze-and-excitation CNN. Wang’s model outperformed other models that used, for example, an SVM, random forest, perceptron [10]. Zuo et al. developed a model based on a spiking neural network (SNN), which also referred to as the third-generation neural network. Zuo extracted features using local mean decomposition (LMD), which is one of the signal processing methods, and encoded the features into SNN. Zuo’s model produces great performance and the capability of SNN for fault diagnosis is confirmed [19].

Despite the remarkable achievement of the studies introduced above, models can only classify the data of the same domain exactly. The domain shift problem occurrs because the distributions of domains are different from each other. Domain adaptation is essential to solve the domain shift problem and it has succeeded in solving this problem in many fields such as classification of text [20] and image [21]. There are two domains in the domain shift problem. One is labeled and used in training for extracting features and the other is unlabeled or partially labeled and cannot be used to train a model for extracting features. For bearings, the domains are varied with experimental environments, bearing models, degree of defects, and the rotational speed, etc. If two datasets are extracted from different domains, the classification boundaries which are trained with one system may fail to classify the states of other bearings. This problem is called a cross-domain fault diagnosis. Much research has been conducted on how to transfer knowledge to solve this problem [22]. Especially, studies of transfer learning with deep learning have rapidly increased in recent years. First, the discrepancy between the two domains has been reduced using some metrics in bearing diagnosis. Lu et al. proposed deep neural network model for domain adaptation to reduce the discrepancy of different domains using MMD [23]. Guo et al. developed deep convolutional transfer learning network which consists of a condition recognition module and a domain adaptation module [24]. For developing a robust method for the noise, Li et al. used a clustering algorithm with a method based on deep learning [25]. Wen et al. made a structure with multi-layer sparse auto-encoder and combined MMD to implement cross-domain diagnosis [26]. Multi-layer MMD was calculated and used in [27]. To improve the accuracy of domain adaptation and obtain stable results, MMD with multi kernel was also reviewed in [28,29,30]. Deep transfer network with joint distribution adaptation for fault diagnosis was studied in [31]. Second-order statistics were used with CNN and utilized for cross-domain fault diagnosis in [32,33]. Wasserstein distance based deep adversarial transfer learning models were developed by Cheng et al. and Zhang et al. [34,35,36]. Second, methods for transferring parameters of the source domain for classification of other domains were studied in [37,38,39,40]. Kim et al. proposed repurposing method for parameter transfer [41]. Third, CNN with an adversarial concept was actively performed [42,43]. Furthermore, Zhao et al. conducted a study to implement and compare the various models and provided the implemented source [44].

All the above studies focus on post-processing methods, but certain domains may not be enough to be accurately adapted. If a suitable signal processing method is implemented, the characteristics of faults can be made easily noticeable, and the training time of the model can be reduced while the accuracy can be improved. Pre-processed signals were used as input for the deep domain generalization network for fault diagnosis (DDGFD) model in [45]. However, prior knowledge such as fault frequencies is required to use this method.

In this paper, using a proposed signal processing method is verified in making all signals into a common pattern space without prior knowledge, such as of the characteristics of faults, and using them is helpful for the cross-domain diagnosis problem. For the study, vibration signals acquired from two different ball bearing systems were analyzed in the time domain and the frequency domain to check whether the fault characteristics can be shown in the signals and the need of pre-processing. Using the proposed method we confirmed that not only do the characteristics of faults appear more clearly, but also signals of different systems are placed in the same pattern space. In addition, the results of classification when using pre-processed datasets are greatly improved compared to using raw data.

This paper is organized as follows: Section 2 introduces preliminary knowledge needed for this paper. The processed method and procedure of making input data are explained in Section 3. Next, the experiment and the results are given with descriptions of the datasets and model used for this paper in Section 4. The conclusion is given in Section 5.

## 2. Preliminary Knowledge of the Paper

### 2.1. Formulation of Cross-Domain Fault Daignosis

There are two domains in cross-domain fault diagnosis. One is source domain, and the other is target domain. All labels of the source domain are defined and available. The expression of source domain is written as follows:

(1) $$D_{s}={\left\{\left(x_{i}^{s},y_{i}^{s}\right)\right\}}_{i=1}^{n_{s}}$$

where, $D_{s}$ is the source domain and $x_{i}^{s}$ is the i-th dataset in the source domain and $y_{i}^{s}$ is the label corresponding to the dataset. Datasets are d-dimensional and the number of datasets is $n_{s}$.

Since the labels of the target domain are not defined or are only defined for some datasets, the labels cannot be used for training extractor. The target domain for which the label is not defined is described as follows:

(2) $$D_{t}={\left\{\left(x_{i}^{t}\right)\right\}}_{i=1}^{n_{t}}$$

where, $D_{t}$ is the target domain and $x_{i}^{t}$ is the i-th dataset in the target domain. As in the source domain, datasets of the target domain are also d-dimensional, and the number of target domain datasets $n_{t}$ may not be equal to $n_{s}$.

Since the source domain and the target domain are extracted from different distributions, there is a high possibility that they will not be classified using the same classification boundaries. Therefore, an appropriate method must be performed to reduce the discrepancy between the source domain and the target domain. That is the goal of cross-domain fault diagnosis.

### 2.2. Convolution Neural Network (CNN)

A Convolutional Neural Network (CNN) is a neural network that employs an operation called convolution instead of general matrix multiplications [46]. CNN is composed of two stages: feature extraction network and classifier network. A feature extraction network, in general, has convolutional layers and pooling layers.

Input data are convoluted with multiple filters (kernels) in convolutional layers. The filters move as much as set stride. The input data are multiplied by trained filters of each convolutional layers, and the convoluted data are extracted as a feature map through an activation function such as ReLU (Rectified Linear Unit).

A certain region which is the size of the pooling is replaced with a representative value in pooling layers so that the size of the feature map is reduced. In general, max pooling and average pooling are used in CNN. Max pooling selects the maximum value, while average pooling averages the values in a specific region.

The convolutional and pooling layers are stacked in the feature extraction network and the final output is the features, also referred to as feature maps. The extracted features go into the fully connected layers of the classifier network, which is basically the same as a standard neural network. The output layer has as many nodes (neurons) as the number of classes, and it is commonly activated by Softmax functions for classification [46]. The loss of classification used in this paper is cross-entropy loss and the models are trained at minimizing it.

### 2.3. Signal Processing

Various filters such as minimum-phase filter and lowpass filter are used to find the characteristics of fault more clearly and all signals are transformed to be located in the same domain using a normal dataset of one system.

Signals are decomposed into minimum-phase system and all-pass system as in Equation (3) in the minimum-phase filter and the part of $H_{min}\left(z\right)$ is used as input data. All zeros and poles of the minimum-phase signal are located inside a unit circle, and this signal is both causal and stable. The group delay of the filtered signal is minimum [47]. The signal processing results of the two data are presented in Section 4.

(3) $$H\left(z\right)=H_{min}\left(z\right)H_{ap}\left(z\right)$$

When the signal is transformed with fast Fourier transform (FFT), spectrum signal is obtained, and the spectrum signal has frequency and amplitude information. The magnitude and phase can be separated by taking a logarithm operation after taking absolute values of FFT signal and a cepstrum domain signal is finally produced when this signal transformed with inverse fast Fourier transform (IFFT). Equation (4) is the equation to obtain cesptrum signal.

(4) $$\hat{x}=F^{-1}\left(\log\left(\left|F\left(x\right)\right|\right)\right)$$

where, $\hat{x}$ is real cepstrum signal, F is FFT and $F^{-1}$ is IFFT, respectively.

In order to obtain a minimum-phase signal, the cepstrum signal is Fourier transformed by covering a window and finally converted to a minimum-phase signal through exponential operating and IFFT. This process can be mathematically expressed as Equation (5) [47].

(5) $$y_{min}=F^{-1}\left(\exp\left(F\left({\hat{x}}_{win}\right)\right)\right)$$

where, $y_{min}$ is minimum-phase signal, ${\hat{x}}_{win}$ is real cepstrum signal, which is covered by a window, respectively.

## 3. Proposed Method

**Figure 1:**
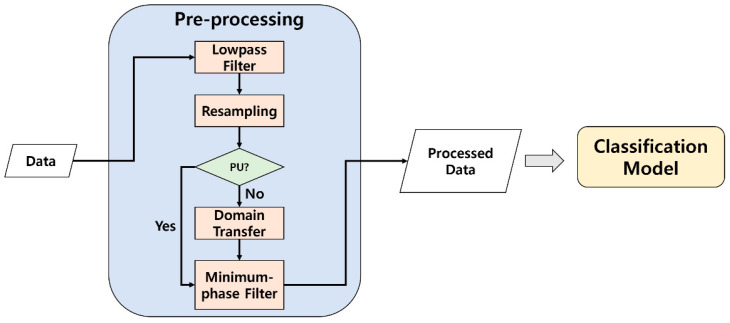
Flowchart of proposed method.

Figure 1 is a flowchart of the proposed method in this paper. To convert signals from different pattern spaces into common space, the signals were pre-processed before entering the classification model. First, a lowpass filter was applied to get rid of noise which was not related to the fault characteristics. Next, since the two systems were collected at different sampling rates, they were resampled with a common rate (4 kHz) by down sampling. Input signals were generated through a minimum-phase filter for the PU datasets. However, for the other dataset rather than PU, such as CWRU, a transfer function was applied. Normal datasets of each system were used to make transfer function. Therefore, for CWRU dataset, the transfer function was applied before the minimum-phase filter to extract the test datasets. By transforming, data of different systems could be located in the same pattern space, and they can be treated altogether.

**Figure 2:**
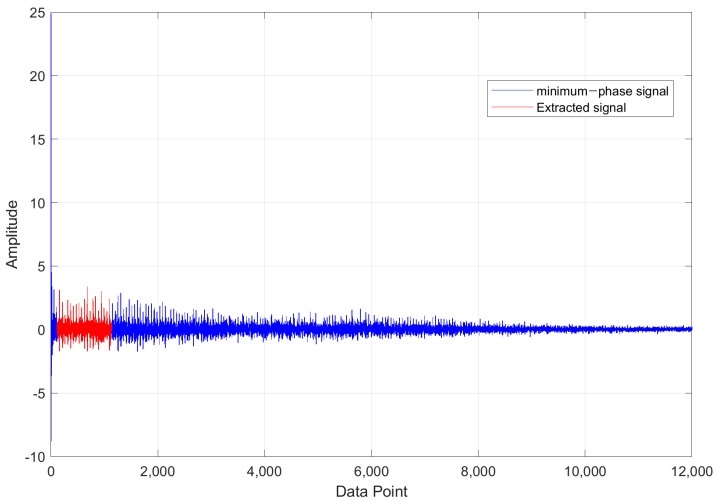
Example of making processed signal.

The input data for the AI model were prepared as follows. For PU datasets, the window was applied to the resampled signal and then the minimum-phase filter was applied. On the other hand, the minimum-phase parts of CWRU data were extracted after applying the transfer function. Figure 2 shows the example of the processed minimum-phase signal (12,000 points of window) and the selected data (shown in red, 1024 points) as the input data. When one dataset was extracted, the window moved by the set value and the next dataset was generated through the same process. 80% of prepared datasets from the source and target domain were used for training and the rest were for testing. For training, labels of the source domain were used while labels of the target domain were not used. In other words, the models were trained with the data from both domains but without labels of the target domain.

Several models which are based in a CNN structure with additional losses or domain adversarial network were compared using raw data and pre-processed data. The classification model was a combination of CNN and domain adaptation methods. The following classification loss was cross-entropy loss which was used for CNN based classification model.

(6) $${𝓁}_{c}=-\overset{C}{\underset{i=1}{\sum }}y^{i}*\log\left({\hat{y}}^{i}\right)$$

where, C is number of class, $y^{i}$ is the actual label and ${\hat{y}}^{i}$ is the predicted output from the CNN. For the domain adaptation, MK-MMD and correlation alignment (CORAL) were used, or domain adversarial network was added. Therefore, the loss (${𝓁}_{D}$) for domain adaptation was combined with classification loss after multiplying trade-off term (λ) as follows:

(7) $${𝓁}_{total}={𝓁}_{c}+\lambda {𝓁}_{D}$$

## 4. Experiments and Results

### 4.1. Datasets and Analyzing of Signals

#### 4.1.1. Case Western Reserve University Data

**Figure 3:**
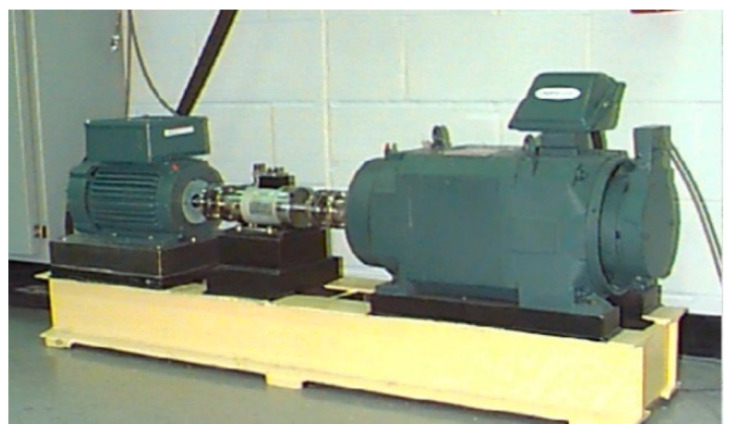
Testbed of CWRU data (electric motor, torque transducer, encoder, and dynamometer and control electronics) [2].

Vibration datasets from two different ball-bearing systems were used for training and testing AI models. The first datasets were from Bearing Data Center of Case Western Reserve University, hereinafter referred to as CWRU data and the testbed for acquiring the dataset was configured as shown in Figure 3. Various conditions were considered in CWRU data such as levels of loads and kinds of defects. There were four load levels from 0 hp to 3 hp in the CWRU datasets. It also contained data with different defect sizes with 0.007 inches, 0.014 inches, and 0.021 inches. The vibration signals were measured by accelerometers with 12,000 sampling rates under four states (normal, outer race fault, inner race fault and ball fault) at various locations. Circular defects were made by electro-discharge machining (EDM). A sampling rate of 48,000 Hz was also set for some cases. Accurate information is provided in [2]. However, only 12,000 Hz drive end bearing datasets with 0.007 inches were used as shown in Table 1.

Before verifying the proposed method, it is important to analyze data in both the time and frequency domains to comprehend the characteristic signals from a defective bearing. Characteristic frequencies of a bearing were computed depending on their specifications [48]. The characteristic frequencies are calculated as follows:

(8) $$BPFO=\frac{nf_{r}}{2}\left(1-\frac{d}{D}cos\emptyset \right)$$

(9) $$BPFI=\frac{nf_{r}}{2}\left(1+\frac{d}{D}cos\emptyset \right)$$

where, *BPFO* is ball pass frequency outer race, *BPFI* is ball pass frequency inner race, $f_{r}$ is the shaft speed, n is the number of rolling elements, d is ball diameter, D is pitch diameter, and ∅ is the angle of the load from the radial plane.

**Figure 4:**
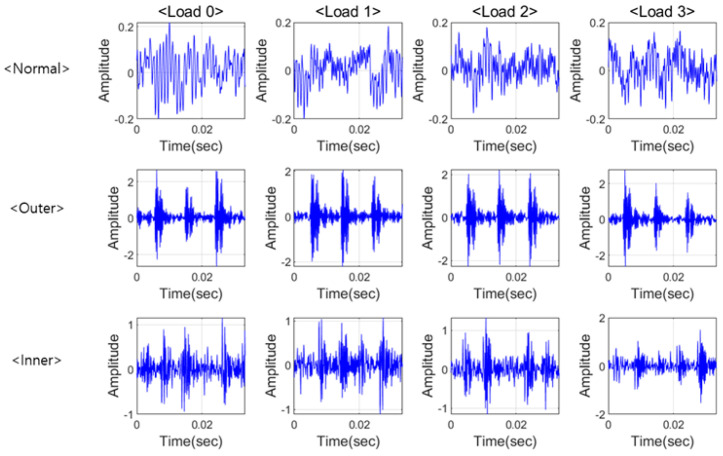
CWRU data in time domain.

**Figure 5:**
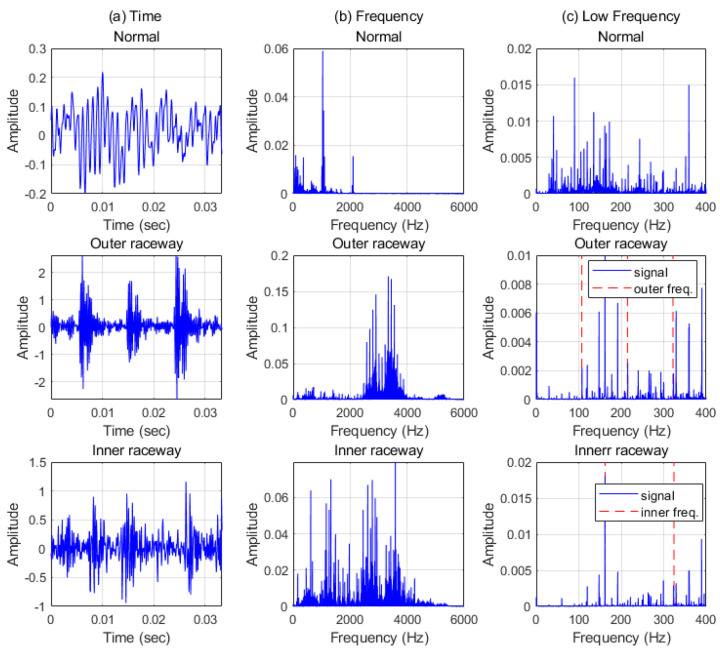
CWRU data with load 0 hp and 0.007 defect in (**a**) time, (**b**) frequency, and (**c**) low frequency. The red dash lines are the characteristic frequencies and their harmonics. BPFO = 107.3 Hz, BPFI = 162.09 Hz.

Data with 0.007 defect and load 0~3 hp are presented in Figure 4 and were transformed with FFT. When bearings have defects on the components such as raceway, physical contacts generate impulse-like signals. Intervals of impulse-like signals represent the characteristic frequencies. Those impulse-like signals also excite the system. Consequently, for all the cases with defects, there is dominant energy in high frequency as shown in Figure 5. Even though there are characteristic frequencies and their harmonics in low frequency, characteristic frequency components are modulated and have more energy in high frequency than in low frequency. However, when there is no impulse-like signal, like the normal case, it is shown that there is no significant energy in high frequency.

#### 4.1.2. Paderborn University Data

**Figure 6:**
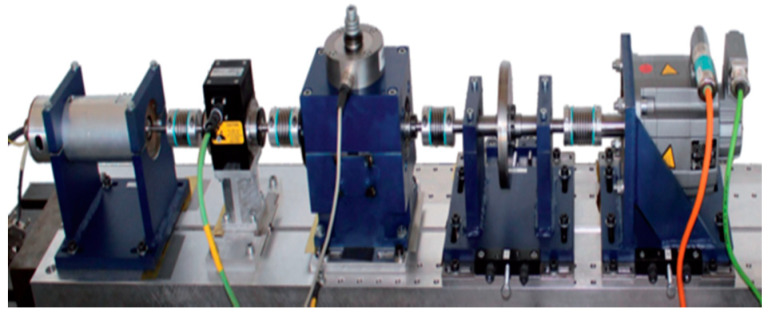
PU data testbed: test motor, measuring shaft, bearing module, flywheel, load motor (from left to right) [1].

The next data are from Konstruktions- und Antriebstechnik datacenter in Paderborn University, hereinafter referred to as PU data and the testbed of the datasets is shown in Figure 6 [1]. PU vibration data were measured by accelerometers with 64,000 sampling rates, and defects were made by using various methods: electric engraver, EDM, drilling, and fatigue. The PU Dataset consists of the signals of healthy bearing (K001~K006), artificial outer raceway faults (KA01, KA03, KA05, KA06, KA07, KA08, KA09), artificial inner raceway faults (KI01, KI03, KI05, KI07, KI08), real outer raceway damage (KA04, KA15, KA16, KA22, KA30), and real inner raceway damage (KI04, KI14, KI16, KI17, KI18, KI21). In addition, the PU data were conducted for various conditions such as rotational speed, load torque, and radial force. Specific information for the datasets is provided in [1]. Table 2 shows the PU dataset which is used in this paper and the description of domain. Datasets of domain P are created using four signals in each of the three states.

**Figure 7:**
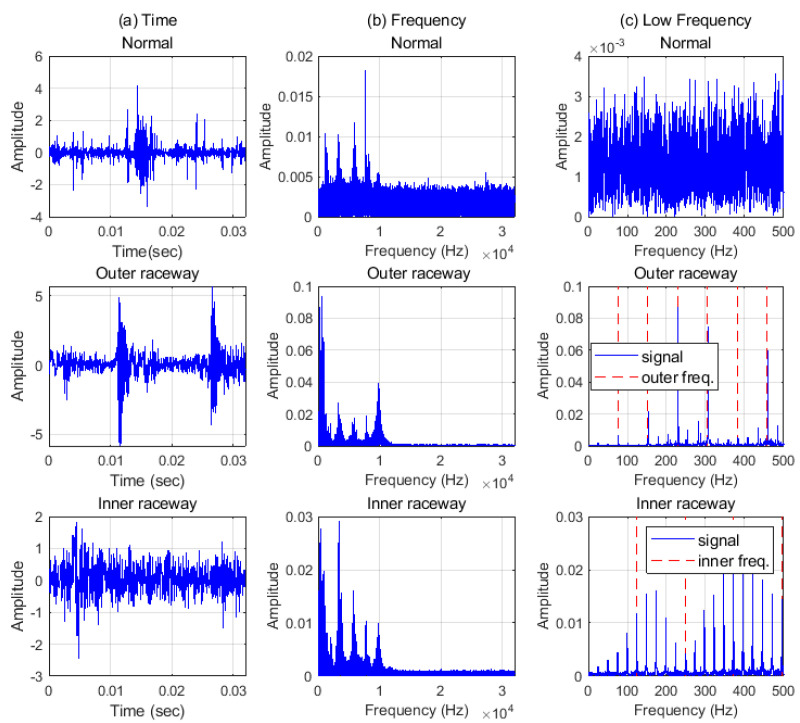
PU data in (**a**) time, (**b**) frequency, (**c**) low frequency domain. The red dash lines are the characteristic frequencies and their harmonics. BPFO = 76.36 Hz, BPFI = 123.64 Hz.

**Figure 8:**
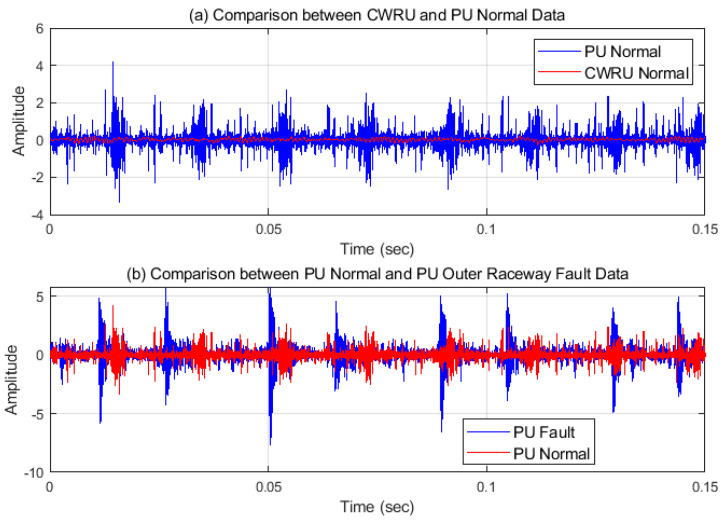
(**a**) Normal signal of CWRU and PU data. (**b**) Normal and outer-raceway fault signal of PU data.

PU data are also analyzed in two domains and some samples of the data are plotted in Figure 7. PU data have more noise than CWRU data and finding the characteristics of inner raceway fault is difficult in the time domain. However, the characteristic frequencies and their harmonics including the rotational components could be found, as shown in Figure 7c. For the PU data, the normal signal has spikes and higher amplitudes than the CWRU normal data has, as shown in Figure 8a, and thus PU normal data could be seemed like a fault signal. However, the PU normal data can be distinguished from the defect data as shown in Figure 8b. This means that defining the normal state is dependent on the person or the experiments and could be different in every case. In other words, the criterion of PU data is more generous than that of CWRU data for the normal state. Therefore, all data are transformed using a PU normal dataset and all features are placed in the same pattern space. In addition, a boundary which is set more generously can classify both domains.

There are distinct characteristic frequencies in the CWRU data and the PU data, but the shapes of spectrums are different according to the system and the fault type. Since the energy of defect characteristic frequencies is very small compared with the total energy, the shape of spectrum is more dependent on the system energy rather than on the defect energy. Only the energy of the characteristic frequencies was studied and used to identify defects. However, information is needed regarding bearings and a system to calculate the characteristic frequencies in advance. Even though the characteristic defect frequencies can be recovered with the traditional signal processing technique, the system and fault characteristics are not known, or the knowledge to obtain them is often insufficient. Hence, if there is a way to transform signals so that fault characteristics can be seen better without prior knowledge, diagnosis can be performed more efficiently. Since the shapes of the signal in the time domain and the frequency domain vary for many reasons other than the defects, the AI models might be trained with other shapes rather than the defect shapes when diagnosis is performed between datasets of different systems. That is why transformation into the common pattern space is necessary. For this purpose, a pre-processing method is proposed, and the results using raw data and pre-processed data are compared and verified. The proposed method not only makes the signals into the same pattern space, but is also effective in removing noise.

### 4.2. Results of Pre-Process

**Figure 9:**
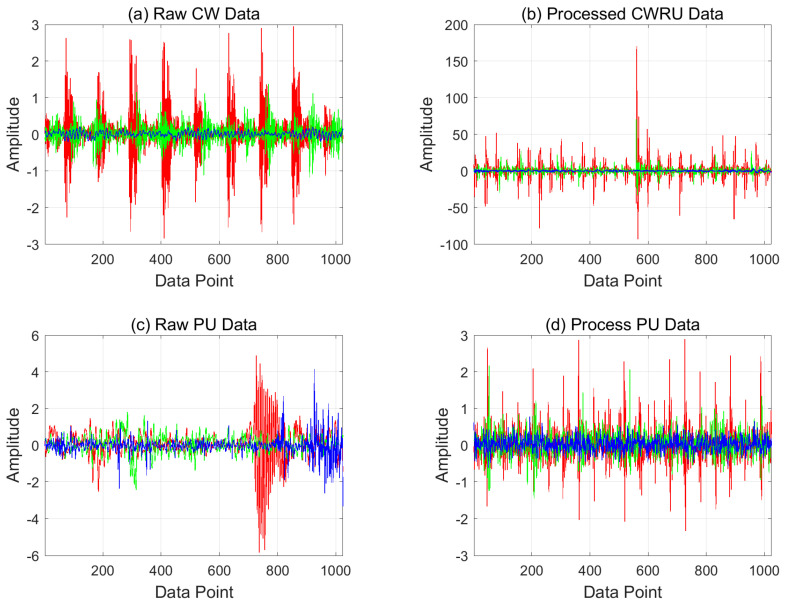
Raw data and processed data: blue lines—normal, green lines—inner raceway fault, red lines—outer raceway fault.

The above analysis of the CWRU and the PU data indicates that it is necessary to transform the two datasets into the same pattern space, and to remove the noise by signal processing. The results of the processed datasets (CWRU and PU) are shown in Figure 9. Each figure shows both the raw and processed signals of the normal and abnormal data. It is shown that raw data have different patterns according to the system while the processed data have similar patterns. In addition, it is not easy to distinguish the states of raw PU data, whereas fault characteristics are more clearly recognizable in processed data as shown in Figure 9d.

### 4.3. Experimental Results and Discussion

Problems were divided into several cases and addressed. Case 1 was conducted using a CNN with pre-processing method to verify the effectiveness of the pre-processing method. In case 2, the ability of the domain adaptation method was examined using raw data and processed data. Finally, the pre-processing method was combined with domain adaptation methods to confirm that the classification accuracies are improved.

#### 4.3.1. Model Description

Basic CNN model is used for classification. Table 3 is the description of basic CNN structure. The model consists of three convolution layers with batch normalization, ReLU, and pooling layers. A fully connected layer was added after extractor. The length of input is 1024 and is labeled as 0 for normal and as 1 and 2 for inner raceway fault and outer raceway fault, respectively. First, CNN was trained as shown in Table 3 for 20 epochs. Next, features extracted from the extractor were trained during 100 epochs to further reduce the difference between the two domains using several methods. The batch size was 64 in both trainings and an Adam optimizer was used. In the first training, datasets and labels of source domain were only used and the target domain data were input without labels in the second training. Training datasets of each domain were 9600 and testing datasets of the target domain were the rest of the datasets of which there were 2368. For case 1, the training epochs were set to 100 without domain adaptation methods and other settings are same as case 2 and case 3.

For the validation of proposed method, several methods which are algorithm based on transfer learning are used as follows:

- CORAL: By aligning the second-order statistics, domain shift between source domain and target domain was minimized [49,50]. For bearing diagnosis, researches were conducted using CORAL in [32,33].
- MK-MMD: MMD was proposed by K.M. Borgwardt et al. [51] and widely used in a cross-domain fault diagnosis for bearing diagnosis [23,24,25,26,27]. The features of the source domain and the target domain were embedded in the reproducing kernel Hilbert space (RKHS), and then the mean distance between the two domains was calculated. By training while reducing this distance, the difference between the two domains was reduced. The MK-MMD method [52] is a method of further reducing domain mismatch by using multi kernel MMD [28,29,30].
- Domain adversarial neural network (DANN): This method was first proposed by Ganin et al. [53] and used in several studies [42,43]. In this method, a discriminator is added, and the features of the source domain and the target domain are not known. For this purpose, a discriminator described in Table 3 was designed and used with gradient reversal layer.

Extractor, classifier, and discriminator were conducted using Python and signal processing and analysis of results were implemented using Matlab. The model is modified and used as demonstrated in [44], with losses. The classification loss was combined with an additional loss multiplied by a trade-off term.

#### 4.3.2. Case 1: CNN with Pre-Processing

**Figure 10:**
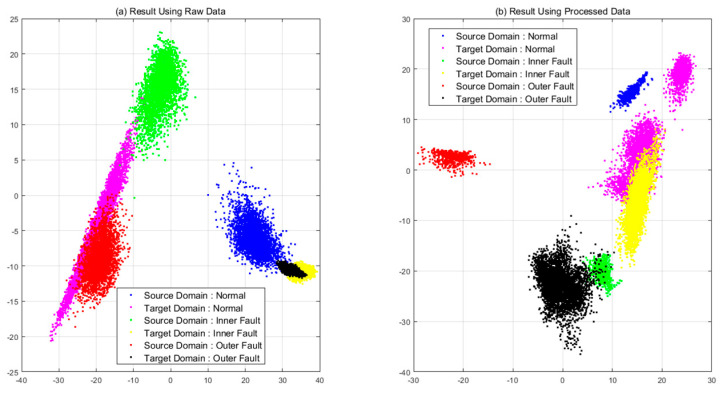
Comparison of features extracted from a model in case 1: (**a**) raw data (**b**) processed data.

CNN with pre-processing was used in case 1. This procedure was conducted to check the effectiveness of the pre-processing method. Table 4 shows the results of classification and Figure 10 the feature distributions in two-dimensional space using principal component analysis (PCA). Features were extracted using raw data located at completely different regions from each other. The processed data show that normal and inner faults are somewhat confused, but features of the defects are abnormal.

#### 4.3.3. Case 2: CNN with Domain Adaptation

**Figure 11:**
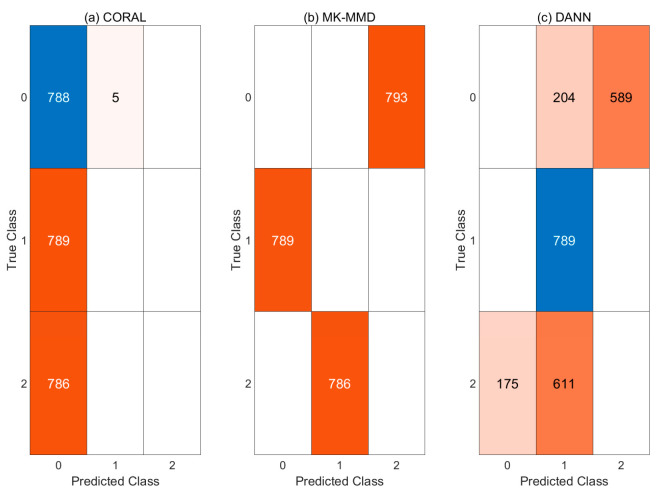
Confusion matrices of case 2: (**a**) CORAL (**b**) MK-MMD (**c**) DANN.

Experiments were conducted without pre-processing in case 2. Table 5 shows the classification results. The highest accuracy of the three models was only 33.32 percent. In the case of the model to which MK-MMD was used, the classification result is zero percent. Confusion matrices for each model are presented in Figure 11.

**Figure 12:**
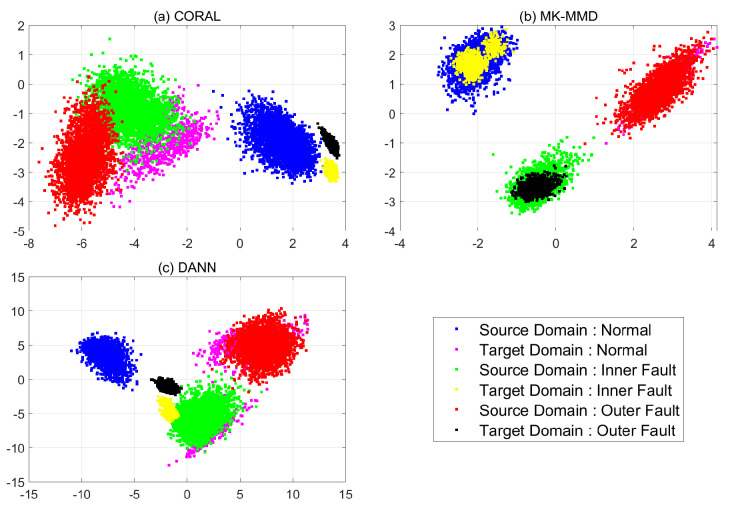
PCA results of case 2: (**a**) CORAL (**b**) MK-MMD (**c**) DANN.

In Figure 12, each feature of three models is compared in the same way as for case 1. Features from the target domain are confused regardless of domain adaptation methods. When CORAL is used, all features of abnormal states are distributed in the normal region of source domain. Therefore, the classification results are all normal. When CNN is combined with MK-MMD, all features of targe domain are placed at totally different states to ther source domain. The features extracted from the CNN which are combined with DANN are not aligned same as when CORAL and MK-MMD are used. Therefore, CNN with pre-processing or post-processing method are helpful for cross-domain problems but the methods are not enough to be used in diagnosis.

#### 4.3.4. Case 3: CNN with Pre-Processing and Domain Adaptation

**Figure 13:**
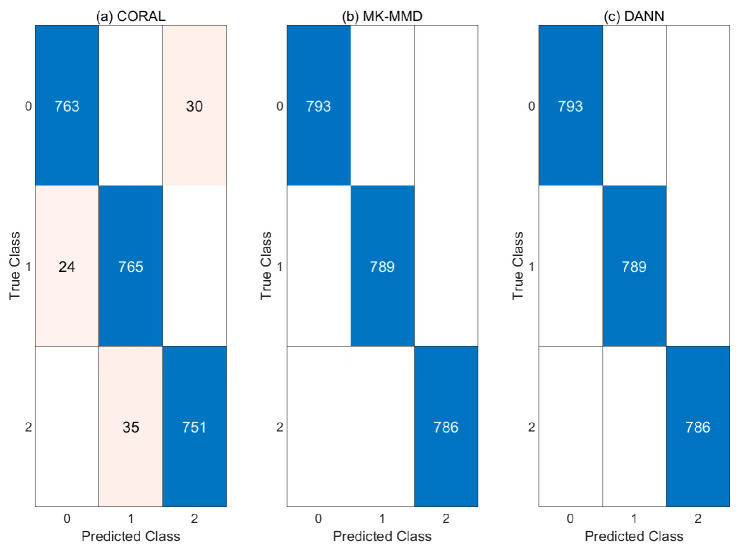
Confusion matrices of case 3: (**a**) CORAL (**b**) MK-MMD (**c**) DANN.

CNN was combined with pre-processing and domain adaptation methods in case 3 and tested. Table 6 shows the accuracies for the case 3. With pre-processing, all the domain adaptation methods were improved compared with case 1 and case 2. Especially, CNN models with pre-processing and MK-MMD or DANN improve classification accuracy up to 100 percent. The confusion matrices of each model are shown in Figure 13.

**Figure 14:**
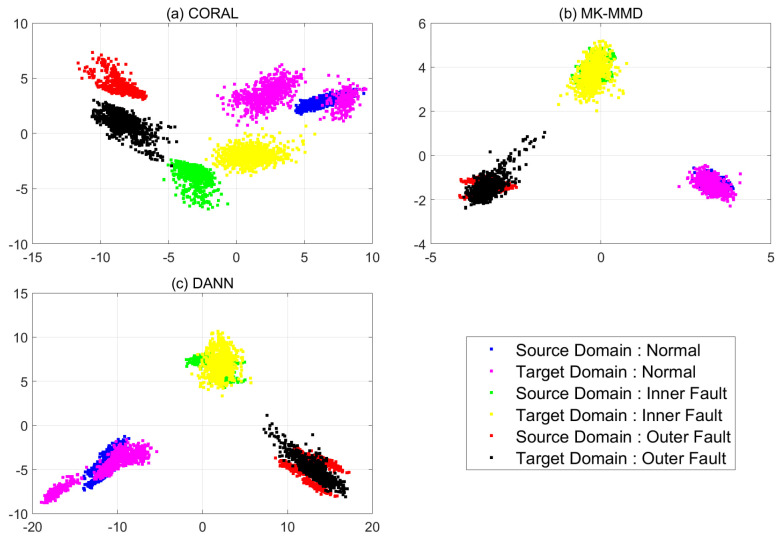
PCA results of case 3: (**a**) CORAL (**b**) MK-MMD (**c**) DANN.

Features extracted from each model were plotted using PCA and the effectiveness of pre-processing is presented as shown in Figure 14. Features extracted using processed data are all located on the same feature space and diagnosis for both systems can be performed using the same decision boundaries. Therefore, the combination of pre-processing and domain adaptation provides quite good performance in cross-domain diagnosing bearing systems.

## 5. Conclusions

Cross-domain fault diagnosis with domain adaptation has shown its good performance in some studies. However, domain adaptation is not enough to deal with the big differences between domains in other cases such as bearing fault diagnosis of different kinds of rotating machines. Therefore, in this paper, a signal pre-processing method was developed to overcome the difficulties in domain adaptation in cross-domain fault diagnosis. The developed pre-processing method was good at not only transforming the signals from different machines into a common domain but also reducing noise to enhance the performance in cross-domain analysis. Unlike other frequency analysis methods, the developed method does not require any prior knowledge such as fault frequencies, which is a great advantage for generalization.

To develop a pre-processing method, two systems (CWRU and PU), which have different specifications of bearings, were analyzed. Various filters and domain transfer methods were reviewed. First, the effectiveness of the pre-processing method was checked. CNN failed to classify the target system and the features of each system turned out to be located at entirely different regions. With pre-processing, accuracy can be improved by 45 percent. Second, CNN with domain adaptation (CORAL, MK-MMD and DANN) was examined. The accuracy was around 30 percent. Both methods were helpful for cross-domain problems but they are not good enough to be used as classifiers. Therefore, pre-processing and domain adaptation method were combined, which improves the accuracy significantly, up to 100 percent. The results were demonstrated and verified with the feature distribution plots using PCA. Therefore, pre-processing with domain adaptation is confirmed to be important for cross-domain fault diagnosis of bearings.

## Figures and Tables

**Table 1:** CWRU dataset used in the paper and domain description.

Types of Faults	Normal	Inner Fault	Outer Fault	Normal	Inner Fault	Outer Fault	Normal	Inner Fault	Outer Fault	Normal	Inner Fault	Outer Fault
Diameter of a defect (Inches)	-	0.007	0.007	-	0.007	0.007	-	0.007	0.007	-	0.007	0.007
Load level	0 hp			1 hp			2 hp			3 hp		
Domain	C1			C2			C3			C4		

**Table 2:** PU dataset used in the paper and domain description.

Bearing Code	Data Number	Rotational Speed (rpm)	Load Toque (Nm)	Radial Force (N)	Types of Faults	Domain
K001	1	1500	0.7	1000	Normal	P
	2					
	3					
	4					
KA16	1	1500	0.7	1000	Outer fault	
	2					
	3					
	4					
KI16	1	1500	0.7	1000	Inner fault	
	2					
	3					
	4					

**Table 3:** Description of basic CNN structure and discriminator.

Role	Layers	Parameters
-	Input	-
Extractor	Convolution 1	Kernel_size = 20, stride = 1, channel = 32
	Batch normalization 1	-
	ReLU 1	-
	Average pooling 1	Kernel_size = 2, stride = 2
	Convolution 2	Kernel_size = 5, stride = 1, channel = 64
	Batch normalization 2	-
	ReLU 2	-
	Average pooling 2	Kernel_size = 2, stride = 2
	Convolution 3	Kernel_size = 3, stride = 1, channel = 128
	Batch normalization 3	-
	ReLU 3	-
	Adaptive average pooling 1	Output size = 4
	Fully connected 1	Out features = 256
	ReLU 4	-
Classifier	Fully connected	Output = 3
Discriminator (for DANN)	Fully connected 1	Out features = 512
	ReLU 1	-
	Fully connected 2	Out features = 1024
	ReLU 2	-
	Fully connected3	Out features = 1
	Sigmoid	-

**Table 4:** Results of case 1.

Domain	Label	Results (%)	
		Raw Data	Processed Data
Train: P	Normal: 0 Inner fault: 1 Outer fault: 2	0.00	45.69
Test: C1–C4			

**Table 5:** Classification results of case 2.

Domain	Label	Model and Results (%)					
		CORAL		MK-MMD		DANN	
		Best	Average	Best	Average	Best	Average
Train: P	Normal: 0 Inner fault: 1 Outer fault: 2	33.28	31.70	0.00	0.00	33.32	21.13
Test: C1–C4							

**Table 6:** Classification results of case 3.

Domain	Label	Model and Results (%)					
		CORAL		MK-MMD		DANN	
		Best	Average	Best	Average	Best	Average
Train: P	Normal: 0 Inner fault: 1 Outer fault: 2	96.24	94.00	100.00	100.00	100.0	100.00
Test: C1-C4							

## Data Availability

The data used to support the findings of this study are publicly available.

## Abbreviations

The following abbreviations are used in this manuscript.

PU	Paderborn University
CWRU	Case Western Reserve University
CNN	Convolution Neural Network
FFT	Fast Fourier Transform
IFFT	Inverse Fast Fourier Transform
CORAL	CORrelation ALignment
MK-MMD	Multi Kernel Maximum Mean Discrepancy
DANN	Domain Adversarial Neural Network
PCA	Principal Component Analysis
